# Comparative evaluation of breathing convenience and clinical acceptance of a new patented mouth breathing device for rubber dam: A randomized clinical study

**DOI:** 10.1016/j.jobcr.2025.11.013

**Published:** 2025-11-28

**Authors:** Geetanjali Jain, Ajay Singh Rao, Shreya Volety, Vidula Bhandary, Rutvi Karande

**Affiliations:** Department of Conservative Dentistry & Endodontics, K. M. Shah Dental College & Hospital, Sumandeep Vidyapeeth (Deemed to be University), Vadodara, 391760, Gujarat, India

## Abstract

**Introduction:**

The rubber dam isolation technique is widely used in endodontics to improve visibility and access, yet it often causes patient discomfort, especially for those with difficulty breathing through their nose. To address this, a new patented mouth breathing device (MBD) was developed to enhance patient comfort by facilitating easier mouth breathing during rubber dam isolation. This study aims to evaluate the breathing convenience and clinical acceptance of the MBD.

**Methods:**

In a randomized clinical trial, 36 patients undergoing endodontic procedures with rubber dam isolation were recruited. Participants were randomly assigned to either the experimental group (MBD) or the control group (no device). A custom-developed questionnaire was used to assess patient comfort, ease of breathing, and overall satisfaction with the procedure. Clinicians also provided feedback on the device's impact on treatment efficiency, patient cooperation, and clinical outcomes. Data were analyzed using statistical tests to compare the results between the two groups.

**Results:**

The study evaluated 36 participants, evenly divided into two groups with a significant age difference (p = 0.04). Group A (Test) showed improved outcomes, including reduced blood pressure (SBP: 14.12 mmHg, DBP: 16.76 mmHg) and increased oxygen saturation (SpO2: +5.71 %, p < 0.001). In contrast, Group B (Control) showed worsened outcomes with increased blood pressure and decreased SpO2 (−6.12 %, p < 0.001).

**Conclusion:**

The patented mouth breathing device offers significant benefits in terms of breathing convenience and patient comfort during rubber dam isolation. The device appears to be a promising adjunct to improve clinical outcomes by reducing patient anxiety and discomfort.

## 1 Introduction

Rubber dam isolation is a gold standard in dentistry, primarily for endodontic and restorative procedures. Introduced by Barnum in 1864, the technique significantly reduces microbial contamination, safeguards patients against aspiration of instruments, and ensures a sterile operating field.^1^^,^^2^, ^3^ Despite its clinical efficacy, the use of rubber dams often evokes discomfort and anxiety among patients, particularly those who rely on mouth breathing. A 2019 global survey revealed that only 44 % of dentists routinely use rubber dams during endodontic treatments, citing patient discomfort as one of the primary deterrents.^4^

Patients unable to breathe comfortably through their nose, due to conditions like nasal congestion or chronic respiratory issues, are at a higher risk of experiencing procedural anxiety. This discomfort can escalate during prolonged dental treatments, leading to interruptions, reduced efficiency, and compromised patient cooperation. The physiological implications are noteworthy, with restricted breathing potentially affecting oxygen saturation and increasing blood pressure during procedures.^5^^,^^6^

Globally, innovations aimed at enhancing patient comfort during dental treatments have gained traction. For instance, studies in the United States and Europe have explored alternatives like adjustable dental chairs and nasal airflow devices.^7^ However, these approaches have limitations, such as increased complexity or cost, and fail to directly address the challenges associated with rubber dam isolation. A systematic review in 2021 emphasized the need for patient-centric solutions, particularly for managing anxiety and breathing difficulties during isolation procedures.^8^

The COVID-19 pandemic further accentuated the importance of rubber dam use. According to a 2020 study published in *The New England Journal of Medicine*, rubber dams significantly reduce aerosol contamination, making them essential in minimizing the transmission of airborne pathogens in dental settings.^9^ However, the pandemic also highlighted the need to address patient discomfort to ensure wider adoption of this critical tool.^10^

To bridge these gaps, a novel, patented mouth-breathing device has been designed to enhance patient comfort during procedures requiring rubber dam isolation. By facilitating unobstructed airflow and reducing procedural anxiety, the device aims to improve patient compliance and procedural outcomes. This randomized clinical study evaluates the effectiveness of this innovative device in comparison to standard rubber dam isolation, focusing on breathing convenience, patient satisfaction, and operator acceptance. The findings could pave the way for more inclusive and patient-friendly practices in global dentistry.

## 2 Methodology

This randomized, double-blinded clinical study was conducted to evaluate and compare the breathing convenience and clinical acceptance of a novel patented mouth-breathing device with standard rubber dam isolation during dental procedures. The research adhered to the guidelines outlined in the Declaration of Helsinki, and informed consent was obtained from all participants. The study adhered to CONSORT guidelines, as shown in the flow diagram (Fig. 3).

**Fig. 1:**
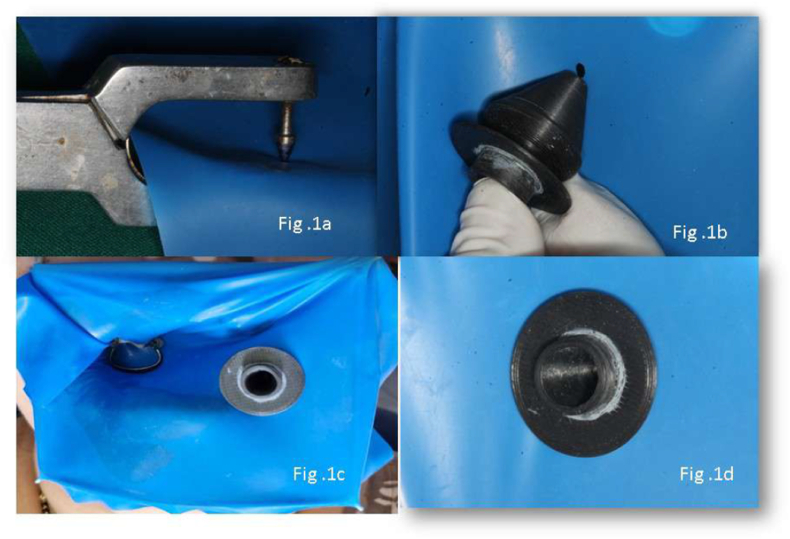
a – punching a hole in the rubber dam. Fig. 1b – inserting the MBD onto the rubber dam. Fig. 1c – rubber dam placed with the MBD on the patient. Fig. 1d - MBD seals the dam from all the sides.

**Fig. 2:**
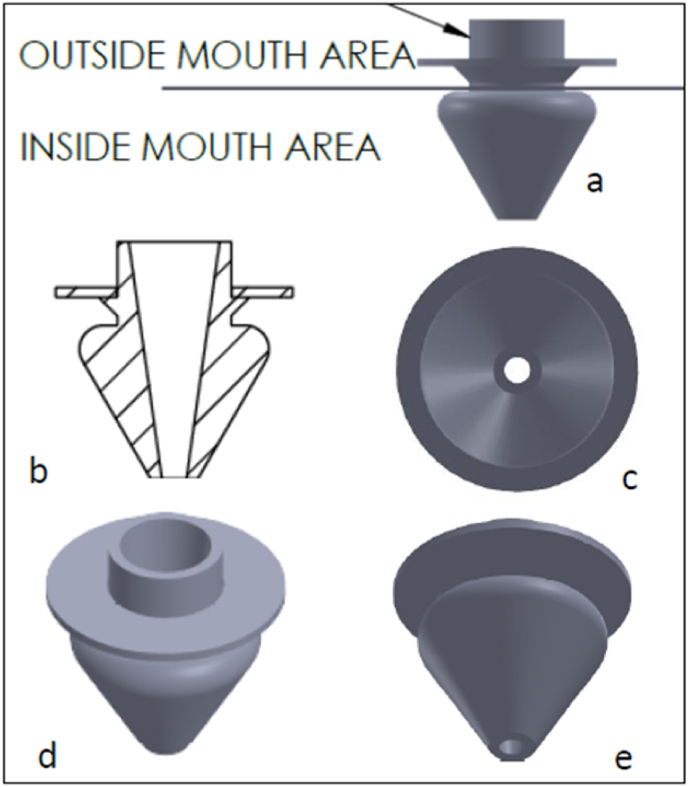
(a–e)- graphical representation of the mouth breathing device.

**Fig. 3:**
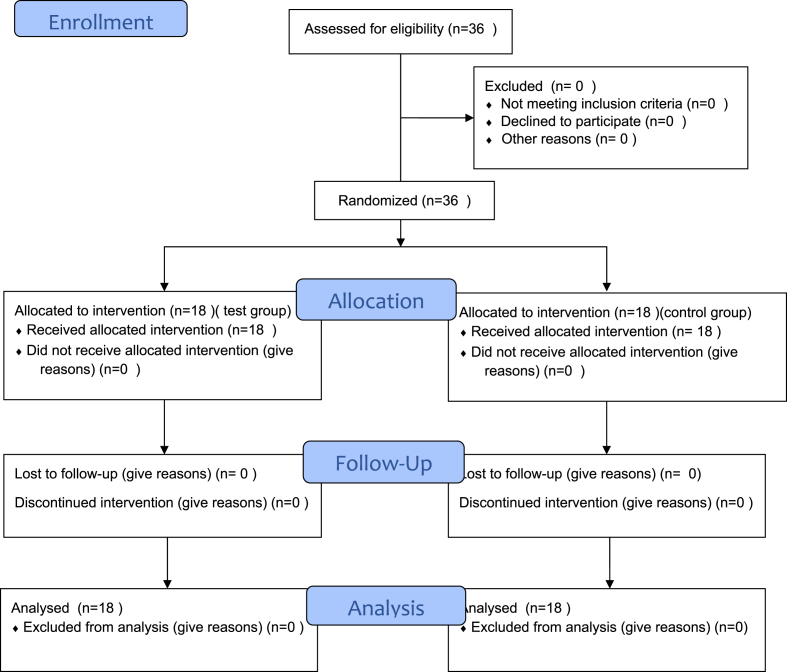
Consort 2010 flow diagram showing participant enrolment, allocation, follow-up and analysis.

**Graph 1:**
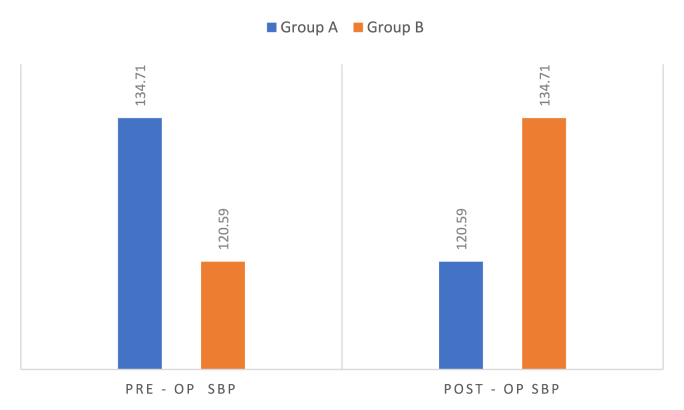
Comparison of pre-op & post-op systolic blood pressure.

**Graph 2:**
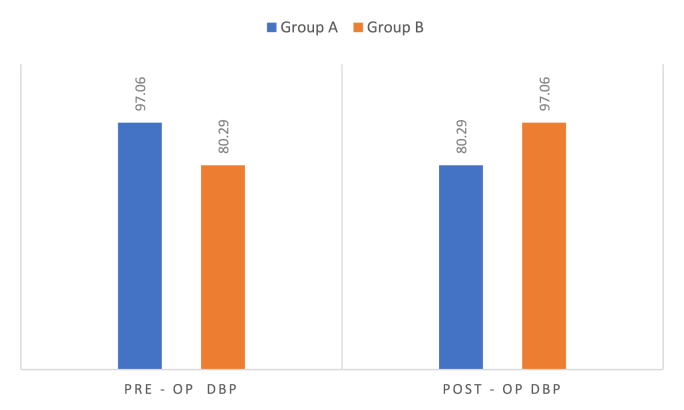
Comparison of pre-op & post-op diastolic blood pressure.

**Graph 3:**
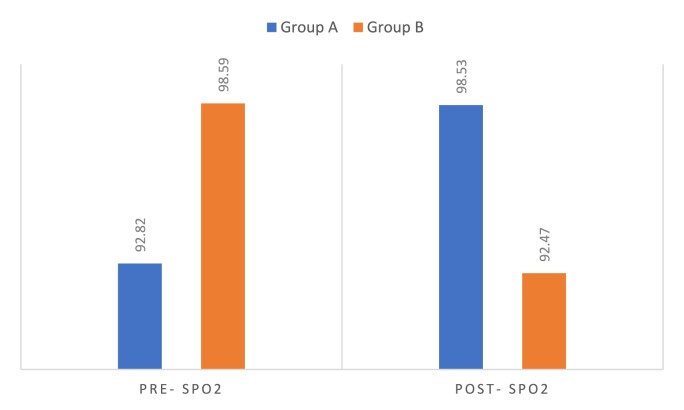
Comparison of pre-op & post-op SpO2.

**Graph 4:**
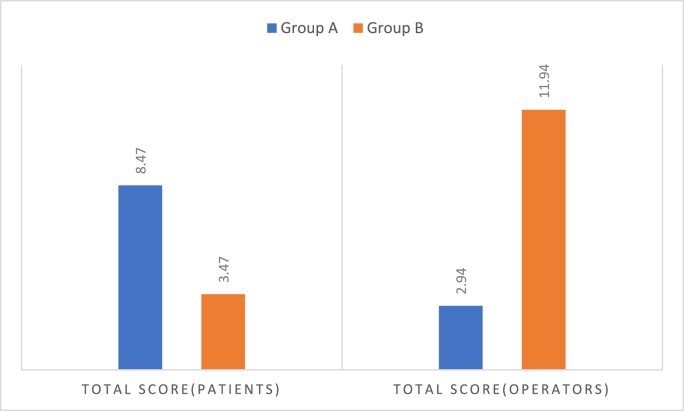
Comparison of pre-op & post-op total score for patients & operators.

The study was carried out at the Department of Conservative Dentistry and Endodontics, K.M. Shah Dental College and Hospital, over a period of 1.5 years. Ethical approval was obtained (SV1EC/ON/Dent/SRP/Oct/24/36), and the study was registered with the Clinical Trials Registry of India (CTRI no- CTRI/2025/02/080196). A pilot study was conducted to determine the sample size, and a final sample size of 36 was obtained for a detailed evaluation followed in the main study.

Participants were selected based on specific inclusion and exclusion criteria.

### 2.1 Inclusion and exclusion criteria

Participants aged 18–60 years with symptomatic irreversible pulpitis in single-rooted teeth, normal periapical tissues, ASA I/II status, and clinically diagnosed mouth breathing were included. Mouth breathing was confirmed using mirror fogging, nasal patency assessment, and clinical observation.^11^ Patients with severe or chronic apical periodontitis, complex canal anatomy, asthma, COPD, systemic illnesses, cognitive impairments, or pregnancy/lactation were excluded. Chronic apical periodontitis was excluded because pre-existing inflammation may alter baseline physiological values and confound outcome measurements.

### 2.2 Design of the MBD

The patented MBD integrates securely with the rubber dam frame and incorporates a dedicated airflow port to facilitate unobstructed mouth breathing without compromising isolation (Fig. 1). It is designed to enhance patient comfort during procedures requiring rubber dam placement, particularly for individuals who experience nasal breathing difficulty. The strategically positioned breathing port allows smooth airflow, helping patients maintain adequate ventilation throughout treatment (Fig. 2).

Ergonomically contoured from biocompatible, sterilizable silicone, the device fits comfortably in the oral cavity without irritating soft tissues or interfering with operator access. Its lightweight, compact structure ensures stability during the procedure while maintaining a sterile, uncontaminated working field. The material's durability allows repeated sterilization without loss of integrity, ensuring safety and hygiene during clinical use.

The device includes safety features to prevent dislodgement during treatment. It is compatible with various rubber dam systems, making it easy to incorporate into standard dental practices without requiring major procedural changes.

Patients were screened for mouth breathing using a standardized test that included clinical observation (such as habitual open mouth and lip posture),^11^ a mirror test to detect fogging during exhalation, and nasal patency assessment. Only those confirmed to exhibit mouth breathing were enrolled in the study. These patients then underwent rubber dam isolation during dental procedures, with the aid of a dedicated mouth breathing device designed to facilitate breathing through the mouth. Their breathing convenience and clinical acceptance of the device were evaluated using a validated questionnaire, while clinicians also provided feedback on the device's impact on the dental workflow and patient management.

### 2.3 Groups

**Test Group (Group A, n = 18):** Rubber dam plus the MBD.

**Control Group (Group B, n = 18):** Rubber dam without any additional device.

Before initiating root canal treatment, patients were seated comfortably, and vital signs, including systolic blood pressure (SBP), diastolic blood pressure (DBP), and heart rate, were recorded using a digital sphygmomanometer (*APOLLO India Pvt Ltd).* Oxygen saturation (SpO2) was measured with a pulse oximeter (*APOLLO India Pvt Ltd*) by Co-Investigator 2. All steps of the root canal treatment were performed by the principal investigator under local anesthesia (1:200,000 Lignocaine with Adrenaline) in a standardized manner. The teeth were isolated using a rubber dam (*SANCTUARY*). Preoperative vital signs were recorded by Co-Investigator 2 before the treatment commenced, and postoperative readings were taken at the end of the procedure and 1 h later.

Eighteen different operators, trained in rubber dam application, were selected, with each operator assigned to one patient. The operators were instructed to place the rubber dam with or without the device, depending on the patient's group assignment. Once the endodontic treatment was completed, both the operator and the patient were provided with a self-designed evaluation criteria proforma.

Self-designed criteria were created as no evaluation criteria were available in the literature. The questionnaire included 10 items evaluating breathing ease, comfort, anxiety, and overall experience. These criteria were validated by three subject specialists, with more than seven years of experience, which were then evaluated by two additional specialists. The evaluation criteria were categorized as follows: 1) Essential, 2) Useful but not essential, and 3) Not essential.^12^ The internal consistency of the responses was validated using the intraclass correlation coefficient (ICC), with a calculated value of 0.840.

### 2.4 Statistical analysis

The data collected were analyzed using IBM SPSS Statistics (Version 22) following CONSORT. Descriptive statistics summarized demographic and clinical variables. The normality of data distribution was assessed using the Shapiro–Wilk test. Independent t-tests were used to compare mean differences between the groups, while paired t-tests were used for intragroup comparisons. Chi-square tests were employed for categorical data. A p-value of <0.05 was considered statistically significant.

All statistical tests were two-tailed, and confidence intervals were calculated at the 95 % level to ensure robustness of comparisons.

## 3 Results

The study evaluated 36 participants, evenly distributed into two groups: Group A (Test Group, n = 18) and Group B (Control Group, n = 18). The mean age of Group A was 36.88 ± 12.26 years (range: 18–63), while Group B had a significantly higher mean age of 45.71 ± 11.78 years (range: 28–70; p = 0.04).

Group A demonstrated significant improvements in physiological outcomes compared to Group B (Table 1).

**Table 1:** Comparison of demographic variables and pre- and post-operative physiological parameters between the test group (MBD) and control group using independent t-tests**.**

	Group A(n = 18)	Group B(n = 18)	t	P VALUE
	Mean ± sd	Mean ± sd		
Age	36.88 ± 12.26	45.71 ± 11.78	−2.14	**0.04**
PRE - OP SBP	134.71 ± 11.08	120.59 ± 4.11	4.927	**<0.001**
POST - OP SBP	120.59 ± 4.11	134.71 ± 11.08	−4.927	**<0.001**
SBP difference	14.12 ± 11.63	−14.12 ± 11.63	7.075	**<0.001**
PRE - OP DBP	97.06 ± 8.1	80.29 ± 3.35	7.889	**<0.001**
POST - OP DBP	80.29 ± 3.35	97.06 ± 8.1	−7.889	**<0.001**
DBP difference	16.76 ± 7.52	−16.76 ± 7.52	12.997	**<0.001**
PRE- spo2	92.82 ± 1.85	98.59 ± 0.51	−12.421	**<0.001**
POST- SPO2	98.53 ± 0.51	92.47 ± 1.84	13.068	**<0.001**
SPO2 differences	5.71 ± 1.9	−6.12 ± 2.06	17.421	**<0.001**
TOTAL SCORE(PATIENTS)	8.47 ± 0.62	3.47 ± 1.01	17.396	**<0.001**
TOTAL SCORE(OPERATORS)	2.94 ± 2.19	11.94 ± 1.56	−13.789	**<0.001**

### 3.1 Systolic blood pressure (SBP)

In Group A, the mean pre-operative SBP was 134.71 ± 11.08 mmHg, which reduced significantly post-operatively to 120.59 ± 4.11 mmHg, yielding a mean reduction of 14.12 mmHg (p < 0.001). In contrast, Group B exhibited an increase in SBP from 120.59 ± 4.11 mmHg pre-operatively to 134.71 ± 11.08 mmHg post-operatively, with a mean increase of 14.12 mmHg (p < 0.001) (Table 1, Table 2)

**Table 2:** Intragroup comparison of pre- and post-operative physiological parameters in the test and control groups using paired t-tests.

			N	Mean ± SD	Mean difference ± SD	t	P VALUE
Group A	Pair 1	PRE - OP SBP	18	134.71 ± 11.08	14.12 ± 11.63	5.00	**<0.001**
		POST - OP SBP	18	120.59 ± 4.11			
	Pair 2	PRE - OP DBP	18	97.06 ± 8.1	16.77 ± 7.52	9.19	**<0.001**
		POST - OP DBP	18	80.29 ± 3.35			
	Pair 3	PRE- spo2	18	92.82 ± 1.85	−5.71 ± 1.9	−12.41	**<0.001**
		POST- SPO2	18	98.53 ± 0.51			
Group B	Pair 1	PRE - OP SBP	18	120.59 ± 4.11	−14.12 ± 11.63	−5.00	**<0.001**
		POST - OP SBP	18	134.71 ± 11.08			
	Pair 2	PRE - OP DBP	18	80.29 ± 3.35	−16.77 ± 7.52	−9.19	**<0.001**
		POST - OP DBP	18	97.06 ± 8.1			
	Pair 3	PRE- spo2	18	98.59 ± 0.51	6.12 ± 2.06	12.26	**<0.001**
		POST- SPO2	18	92.47 ± 1.84			

### 3.2 Diastolic blood pressure (DBP)

In Group A, DBP decreased from 97.06 ± 8.10 mmHg to 80.29 ± 3.35 mmHg, with a mean reduction of 16.76 mmHg (p < 0.001). However, Group B showed an increase from 80.29 ± 3.35 mmHg to 97.06 ± 8.10 mmHg, with a mean increase of 16.76 mmHg (p < 0.001) (Table 2).

### 3.3 Oxygen saturation (SpO2)

Group A exhibited improved oxygenation, with mean SpO2 increasing from 92.82 ± 1.85 % pre-operatively to 98.53 ± 0.51 % post-operatively (mean increase: 5.71 %; p < 0.001). Conversely, Group B experienced a decline in SpO2, dropping from 98.59 ± 0.51 % pre-operatively to 92.47 ± 1.84 % post-operatively (mean reduction: 6.12 %; p < 0.001). This demonstrates that the test group experienced reduced stress and improved airway stability during the procedure (Table 2).

### 3.4 Patient & operator feedback

Patient comfort scores were significantly higher in Group A (8.47 ± 0.62) vs. Group B (3.47 ± 1.01; p < 0.001). Operators also reported improved workflow and fewer interruptions in Group A (2.94 ± 2.19 vs. 11.94 ± 1.56; p < 0.001).

These subjective outcomes align with physiological improvements.

## 4 Discussion

This study is one of the first clinical evaluations of a dedicated mouth-breathing device (MBD) designed to improve patient comfort and physiological stability during rubber dam isolation. Mouth breathing is known to disturb normal respiratory mechanics and is associated with shallow breathing, altered oxygen–carbon dioxide balance, and increased sympathetic activation. These changes can trigger anxiety-related symptoms such as tachycardia, muscle tension, and dizziness, contributing to patient stress during dental procedures.^13^ Fitzpatrick et al. further demonstrated that the route of breathing directly influences upper airway resistance, with oral breathing increasing respiratory effort and potentially exacerbating stress responses.^14^, ^15^ These findings have particular relevance in dental settings, where patients are already prone to anxiety.

In contrast, nasal breathing supports stable ventilation and promotes parasympathetic activation, which enhances relaxation and reduces physiological stress. When nasal airflow is limited—as commonly seen in habitual mouth breathers or patients with nasal obstruction—rubber dam placement can intensify discomfort by further restricting airflow. Our study demonstrates that the MBD helps alleviate this problem by providing an unobstructed oral airway, thereby reducing the breathing-related stress commonly reported during isolation procedures.

Breathing difficulties during dental treatment can have measurable physiological consequences. Previous studies have shown that altered breathing patterns can reduce oxygen saturation and increase cardiovascular strain ^6^,^16^, ^17^. In our study, Group B (rubber dam alone) showed a significant reduction in SpO_2_ and an increase in SBP and DBP, supporting earlier findings that mouth-breathing under stress may compromise oxygenation and elevate sympathetic output. Conversely, Group A demonstrated an improvement in SpO_2_ and reductions in SBP and DBP, indicating that the device helped maintain airway patency, prevent hypoxia-related stress, and stabilize physiological parameters.^17^, ^18^

Johnson et al. reported that oxygen desaturation during oral breathing can impair cognitive function and increase the physiological burden on patients.^17^ Similarly, Niaki et al. observed reduced oxygen saturation in mouth-breathing individuals compared to nasal breathers.^6^ The present study's findings align with these results, showing clear physiological advantages when the airway is supported by the MBD.

Patient and operator feedback further reinforces these physiologic outcomes. Patients using the device reported significantly greater comfort and reduced anxiety, while operators noted fewer interruptions, improved cooperation, and smoother workflow. These practical benefits are clinically meaningful, as discomfort and anxiety are among the most frequent reasons for patients refusing rubber dam isolation.^4^^,^^19^ By improving breathing comfort, the device may help increase patient acceptance of rubber dams, addressing a long-standing barrier to their routine use.

In addition to improving comfort, optimized breathing may reduce adverse oral effects associated with chronic mouth breathing. Studies have shown that persistent mouth breathing is linked to oral dryness and an increased risk of dental caries due to reduced salivary flow.^6^ Therefore, facilitating proper airflow during dental procedures may indirectly contribute to better oral health by minimizing these effects.

Overall, the results of this study demonstrate that the MBD significantly enhances patient comfort, improves physiological stability, and supports clinicians by reducing procedural challenges. The device shows promise as a valuable adjunct for improving patient experience and clinical efficiency during rubber dam-based procedures.

## 5 Limitations

Our study has limitations, including the relatively homogenous patient population and short-term design. Future studies could explore the device's impact on diverse patient demographics and during extended or more complex procedures. Investigating long-term outcomes, such as sustained reductions in patient anxiety and improved procedural efficacy, would also provide valuable insights.

## Funding

This is a self-funded study without any external funding done for the same.

## Declaration of Competing interest

Dear Editor-in-Chief, I am writing to formally submit my manuscript titled “Comparative Evaluation of Breathing Convenience and Clinical Acceptance of a New Patented Mouth Breathing Device for Rubber Dam: A Randomized Clinical Study” for consideration for publication in the Journal of Oral Biology and Craniofacial Research. This manuscript represents original research conducted by my co-authors and me, and we believe it contributes valuable insights to the field of oral biology and craniofacial research.

I hereby declare that there are no conflicts of interest associated with this manuscript. All authors have contributed significantly to the work, reviewed the final manuscript, and approved its submission to your esteemed journal. Additionally, this manuscript has not been published elsewhere, nor is it under consideration for publication in any other journal.

We confirm that ethical guidelines have been followed, and any necessary institutional approvals have been obtained. Any funding sources or financial support for this research have been disclosed within the manuscript.

We sincerely appreciate your time and consideration, and we look forward to the opportunity to contribute to the journal. Please do not hesitate to contact me should you require any further information.
